# ADRB3 polymorphism rs4994 (Trp64Arg) associates significantly with bodyweight elevation and dyslipidaemias in Saudis but not rs1801253 (Arg389Gly) polymorphism in ARDB1

**DOI:** 10.1186/s12944-018-0679-7

**Published:** 2018-03-27

**Authors:** Maha Daghestani, Mazin Daghestani, Mamoon Daghistani, Abdelmoneim Eldali, Zeinab K. Hassan, Maha H. Elamin, Arjumand Warsy

**Affiliations:** 1 Department of Zoology, Center for Scientific and Medical Female Colleges, King Saud University, P.O. Box 22455, Riyadh, 11495 Saudi Arabia; 2 Department of Obstetrics and Gynecology, Umm-Al-Qura University, P.O.Box 424, Makkah, 21955 Saudi Arabia; 3 Department of Surgery, King Abdulaziz Medical City, National Guard Health Affairs, P.O.Box, Jeddah, 9515 Saudi Arabia; 4 Department of Biostatistics, Epidemiology and Scientific Computing, King Faisal Specialist Hospital and Research Center, P.O. Box3354, Riyadh, 11211 Saudi Arabia; 5 Virology and Immunology Unit, Cancer Biology Department, National Cancer Institute Cairo University, Cairo, Egypt; 6 Department of Zoology, Faculty of Sciences, King Saud University, P.O. Box 22452, Riyadh, 11495 Saudi Arabia; 7 Senior Scientist, Central Laboratory, Center for Scientific and Medical Female Colleges, King Saud University, P.O. Box 22455, Riyadh, 11495 Saudi Arabia

**Keywords:** β1 adrenoceptor, β3 adrenoceptor, Dyslipidaemias, Polymorphism, Obesity, Saudi population, χ^2^ Chi square

## Abstract

**Background:**

In some populations, obesity and body weight related disorders show a correlation with polymorphisms in three subtypes of beta-adrenoceptor (β1, β2, and β3) [*ADRB1, ADRB2* and *ADRB3]* genes. We scanned for the polymorphism of Arg389Gly (rs1801253**)** in *ADRB1* and Trp64Arg (rs4994) in *ADRB3* genes in Saudi population to determine association, if any, of these polymorphisms with obesity and related disorders.

**Methods:**

We studied 329 non-related adults (33.1% men and 66.9% women), aged 18–36 years. Anthropometric measurements were recorded, and Body mass index (BMI) and waist/hip ratio were calculated; leptin, insulin, lipidogram, and glucose concentrations were determined. *ADRB1* and *ADRB3* polymorphisms (Arg389Gly and Trp64Arg, respectively) were screened by DNA sequencing. The subjects were divided into three groups according to BMI: normal weight (BMI < 25 kg/m^2^), overweight (BMI ≥25.1–29.9 kg/m^2^) subjects, and obese (≥30 kg/m^2^).

**Results:**

In the age-matched groups of the normal weight, overweight and obese male and female subjects, all anthropometric parameters were found to be significantly higher, and in the obese group, all biochemical parameters were significantly elevated compared to the normal weight controls. The allelic frequency of Gly389 *ADRB1* did not differ amongst the three groups, whereas the frequency of Arg64 of *ADRB3* gene was significantly higher in the overweight and obese subjects, compared with the normal weight subjects. In addition, subjects carrying Arg64 allele regardless of their BMI had a greater waist and hip circumference, W/H ratio, plasma cholesterol, triglyceride, LDL, leptin, insulin, and glucose level compared to those with the wild-type Trp allele.

**Conclusion:**

The results of this study have shown a significant association between the Trp64Arg polymorphism in *ADRB3* gene and the development of overweight and obesity in Saudi populations. It also has an influence on the levels of lipid, insulin, leptin, and glucose, whereas, Arg389Gly polymorphism in *ADRB1* is not associated with overweight, obesity or dyslipidaemias in Saudis.

## Background

Obesity and diabetes mellitus Type 2 (T2DM) are rapidly growing public health problems in the Saudi population [1, 2]. A high risk of T2DM and cardiovascular complication is also associated with increased obesity [2, 3]. Several epidemiological and clinical studies have shown that human obesity is a multifactorial disorder, with both genetic predisposition and environmental factors contributing to its etiology [4, 5]. Extensive studies have linked different genetic loci to obesity, and strong linkage has between reported between beta (β) adrenoceptor polymorphisms (ADRB) and obesity or weight gain [6–10]. There are three subtypes of ARDB: β-1, β-2, and β-3, and these are involved in development, behavior, smooth muscle tone, heart function, and energy metabolism [11]. The three β -subtypes (β-1, β-2, and β-3) coexist in both white adipose tissue (WAT) and brown adipose tissue (BAT). In WAT, ADRB signaling is thought to stimulate lipolysis in response to fasting, whereas in BAT, it mediates heat production in response to cold exposure or overfeeding via activation of the uncoupling protein-1(UCP1) [12–14]. Catecholamines are regulators of lipolysis, and act via β1-, β2-, β3- (stimulatory), and α2- (inhibitory) adrenoceptor subtypes in adipose tissue. Most of the sympathetic nervous system-mediated energy expenditure in skeletal muscles takes place via the coupling of catecholamines with β2-adrenoceptors [15, 16] and plays important roles in energy expenditure and control of body weight [9–17]. Recently, it has been shown that *ARDBs* are polymorphic with single nucleotide polymorphism (SNPs) exerting functional consequences affecting receptor activity and regulation, and hence may contribute to the pathophysiology of obesity and related disorders [7–9]. Several studies have shown an association between *ADRB* polymorphism and BMI, T2DM, hypertension [HT] and dyslipidaemias, while other studies have failed to show such an association [18–53].

In our previous report, we have shown a link between obesity-related disorders in Saudi population and Gln27Glu polymorphism in *ARDB2* gene [9]. In the present study, we have investigated the relationship between polymorphism in *ARDB1* (rs1801253) and *ARDB3* (rs4994) genes and obesity and related disorders in the Saudi population.

## Methods

### Study group

The study was approved by the local ethics committee at the Umm Al Qura University, Makkah Al Mukaramah, Saudi Arabia (IRB No. 235). It included randomly chosen 329 unrelated Saudi subjects [men = 109 (33.1%) and women = 220 (66.9%)], from a nationwide population, with ages ranging from 18 to 36 years. Informed written consent was obtained from all study subjects before their participation.

### Anthropometric and biochemical measurements

Anthropometric measurements included the recording of height, weight, waist and hip circumference by standard methods. Body mass index (BMI; kg/m^2^) and waist-to-hip ratio (WHR) were calculated [54]. For biochemical studies, 20 ml of blood was drawn by venipuncture from an antecubital vein without compression, following an overnight fast, from each subject under study. 10 ml blood was placed in ethylenediaminetetraacetic acid (EDTA) coated tubes, 2 ml in tubes containing fluoride and the rest in plain tubes. All samples were immediately centrifuged at room temperature to collect the plasma/serum. Plasma glucose was determined in duplicate by a glucose-oxidase method adapted to an autoanalyzer (Hitachi 704, Boehringer Mannheim, Germany). Enzymatic methods using commercial kits (Boehringer Mannheim) were used to estimate total serum cholesterol, triglycerides, low-density lipoprotein [LDL] and high-density lipoprotein [HDL]. Plasma insulin and leptin concentrations were estimated by radioimmunoassay (RIA) using Human Insulin Specific RIA kit and Human Leptin RIA Kit, respectively (Linco Research, St Louis, MO).

### Genotyping of *ADRB1* polymorphism (rs1801253**)**

The genomic DNAs of all the subjects were extracted from peripheral blood leukocytes using Gentra Systems Kit (Minneapolis, MN, cat # D5500). The DNA fragment containing codon 389 of the *ARDB1* gene was amplified by polymerase chain reaction (PCR) using a sense primer (5’-CTCTTCGTCTTCTTCAACTGGCT-3′) and an antisense primer (5’-CAACAAGGAACATCAGCAAGC-3′). The PCR conditions consisted of an initial denaturation step at 95 °C for 15 min, followed by 34 cycles of denaturation at 95 °C for 1 min, annealing at 60 °C for 1 min, and extension at 72 °C for 1 min, with a final extension of 10 min at 72 °C. With these primers, a PCR product was verified on 2% agarose gel electrophoresis. Nucleotide sequencing was carried out by the ABI Big Dye Terminator protocol using ABI 3100 Avant Genetic Analyzer.

### Genotyping of *ADRB3* polymorphism (rs4994)

The DNA fragment containing codon 64 of the *ADRB3* gene was amplified by PCR using a sense primer (5′- CCAGTGGGCTGCCAGGGG-3′) and an antisense primer (5′- GCCAGTGGCGCCCAACGG -3′). The PCR conditions were also the same as mentioned above for *ADRB1* studies. Nucleotide sequencing was carried out by the ABI Big Dye Terminator protocol using ABI 3100 Avant Genetic Analyzer.

### Statistical analysis

Stat View program for Windows (version 8.0; SAS) was used to conduct all data analyses. Based on the value of BMI, the total population was grouped as normal weight (BMI < 25 kg/m^2^), overweight (BMI ≥25.1–29.9 kg/m^2^), and obese (≥30 kg/m^2^). The data obtained was analyzed separately for each group and is presented as mean ± SEM. The comparisons of anthropometric parameters and biochemical and hormonal variables between overweight, obese and normal weight control subjects were carried out using the independent student’s t-test and ANOVA.

The data were further grouped according to the three genotypes of *ARDB1* and *ARDB3* genes, and the anthropometric measurements, age, BMI, fasting serum insulin, leptin, glucose level and lipid profile, were compared by the Mann-Whitney U- test. To study the influence of the *ARDB1* and *ARDB3* genotypes on BMI, multivariable logistic regression was used. The different parameters were correlated using Pearson’s correlation coefficient (r), and the *p*-value was obtained. Frequency distribution analysis was performed using the chi-square test, and frequencies of the different alleles in different groups (normal, overweight and obese) were compared. Relative risk was estimated by the odds ratios (ORs) and their 95% confidence intervals (CIs), and a p-value ≤0.05 was considered statistically significant.

## Results

### Obesity-related anthropometric and biochemical characteristics

Using a BMI cut-off point of BMI < 25 kg/m^2^, BMI ≥ 25.1–29.9 kg/m^2^) and BMI > 30 kg/m^2^ for normal weight, overweight and obese, as recommended by the World Health Organization [55] there were 115, 68, 146 individuals in the normal weight, overweight and obese groups, respectively. The anthropometric characteristics of the study subjects are presented in Table 1. All the three groups matched in their age and no statistically significant differences were observed amongst the groups. All other parameters except HDL, were significantly higher in overweight and obese subjects as compared to the normal weight (control) group (*p* < 0.0001) as shown in Table 1. The results of biochemical and hormonal parameters are presented in Table 2 for the three groups. The value for each parameter was elevated significantly, in the overweight and obese subjects, except HDL, which was significantly lower when compared to the normal weight group (Table 2).

**Table 1:** Anthropometric characteristics of control, overweight and obese study subjects

VARIABLES	Control (*n* = 115) mean ± SEM	Overweight (*n* = 68) mean ± SEM	Obese (*n* = 146) mean ± SEM	*P* value
Age (yr)	24.90 ± 0.44	25.56 ± 0.54	26.70 ± 0.43	0. 056
BMI (kg/m^2^)	21.33 ± 0.19	26.93 ± 0.17	34.89 ± 0.50	0.0001
Waist (cm)	70.82 ± 0.79	85.13 ± 0.93	103.72 ± 1.23	0.0001
Hip (cm)	96.13 ± 0.68	102.22 ± 0.82	119.68 ± 1.15	0.0001
W/H Ratio	0.73 ± 0.006	0.84 ± 0.006	0.87 ± 0.006	0.0001

Values are mean ± SEM. Abbreviations: BMI, body mass index; W/H, waist/hip ratio; SEM: Standard Error of the Mean

**Table 2:** Comparisons of clinical parameters amongst control, overweight and obese study subjects

VARIABLES	Control (*n* = 115) mean ± SEM	Overweight (*n* = 68) mean ± SEM	Obese (*n* = 146) mean ± SEM	*P* value
Cholesterol (mmol/L)	3.43 ± 0.04	4.19 ± 0.09	4.4 ± 0.07	0.0001
Triglyceride (mmol/L)	0.74 ± 0.02	1.082 ± 0.05	1.302 ± 0.04	0.0001
HDL (mmol/L)	1.32 ± 0.03	1.19 ± 0.04	1.07 ± 0.024	0.0001
LDL -cholesterol (mmol/L)	1.55 ± 0.05	2.07 ± 0.89	2.56 ± 0.06	0.0001
Leptin (ng/ml)	9.42 ± 0.48	17.51 ± 0.97	36.13 ± 1.67	0.0001
Fasting Insulin (pmol/L)	57.21 ± 2.09	72.23 ± 4.28	106.03 ± 3.07	0.0001
Fasting Glucose (mmol/L)	4.71 ± 0.041	5.051 ± 0.057	5.06 ± 0.05	0.0001

Values are mean ± SEM. *Abbreviations*: Values are mean ± SEM. *Abbreviations*: *HDL*: High-density lipoproteins; *LDL*: Low-density lipoprotein; *SE*: Standard Error of the Mean

### Polymorphism in *ADRB1* Arg389Gly genotype

The genotype and allele frequencies of the *ADRB1* Arg389Gly (rs1801253**)** polymorphism were calculated in each group and results were compared. There was no significant difference in the genotype and allelic frequency of *ADRB1* Arg389Gly between control and obese or overweight subjects, as shown in Table 3. The *ADRB1* genotypes (CC, CG, GG) were grouped separately, and the phenotypic characteristics, biochemical and hormonal parameters were calculated separately for each genotype. No significant differences were observed in the results obtained for the three genotypes (Table 4).

**Table 3:** Distribution of the genotypes, allele’s frequencies and odd ratio of the ADRB1 Arg389Gly polymorphism in control, overweight and obese subjects

Genotype	Control T = 115	Overweight T = 68	Obese T = 146	χ2 Odds ratio (95% CI)	*P*- value
Genotype Frequency					
Arg\Arg	20 (17.4)	12 (17.6)	18 (12.3)	χ2 Ow = 0.08 Ob = 0.7 *-Odds ratio (95% CI) Control* vs *OW 1.09(0.6–1.9)* -*Odds ratio (95% CI) Control* vs *Obese 1.2(0.75–1.3)*	*P value for Odds ratio in control* vs *overweight: 0.8 and control* vs *obese:0.4*
Arg\Gly	45 (39.1)	25 (36.8)	57 (39)		
Gly\Gly	50 (43.5)	31 (45.6	71 (48.6)		
Allele Frequency					
Allele Arg389	37.0%	36%	31.8%	*Odds ratio (95% CI) Control* vs *OW* 1.04 (0.67–1.6) *Odds ratio (95% CI) Control* vs *Obese1.25(0.9–1.8)*	*P* = 0.86
Allele Gly 389	63.o%	64%	68.2%		*P* = 0.22

OW = Overweight; Ob = Obese; CI: Confidence interval; χ^2^: Chi square

**Table 4:** Phenotypic characteristics of subjects grouped according to ADRB1 polymorphism at Codon 389

Phenotypic variables	Arg\Arg (*n* = 50) *mean ± SE*	Arg\Gly (*n* = 127) *mean ± SE*	Gly\Gly (*n* = 152) *mean ± SE*	*P*-value
BMI (kg/m^2^)	28.563±1.169	28.500±.64	28.489±.5901	0.998
Waist(cm)	87.84±2.706	88.67±1.640	88.31±1.528	0.964
Hip(cm)	107.24±2.343	108.33±1.34	107.63±1.195	0.888
W/H ratio	.8162±.012	.8160±.009	.8145±.008	0.989
Cholesterol (mmol/L)	3.936±.1241	4.016±.0740	4.043±.0720	0.749
Triglyceride (mmol/L)	1.056±0.0680	.078±0.0459	1.048±0.034	0.865
HDL (mmol/L)	1.1986±0.0441	1.1459±0.0275	1.2084±0.0299	0.292
LDL (mmol/L)	2.072±.111	2.094±0.067	2.122±0.068	0.918
Leptin (ng/ml)	19.57±2.149	23.49±1.67	23.60±1.584	0.383
Fasting Insulin (pmol/L)	79.0±5.68	84.21±3.33	81.09±3.26	0.677
Fasting Glucose(mmol/L)	4.900±0.07	5.002±0.050	4.9±0.043	0.203

Values are mean ± SEM. *Abbreviations*: *BMI*: body mass index; *W/H*: waist hip ratio; High-density lipoproteins; *LDL*: Low-density lipoprotein; *SEM*: Standard Error of the Mean

### Polymorphism in ADRB3 Trp64Arg genotype

The frequencies of Trp64Arg (rs4994) alleles and genotypes were calculated. The overweight and obese subjects had a significantly higher genotype and allele frequency of Arg64 compared with normal weight subjects. The genotype and allele frequencies in the total group, are presented in Table 5 with the OR, CI, χ^2^ and *p*-value. Furthermore, the study groups were separated according to their Trp64Arg genotypes (TT, TC, CC), and the phenotypic characteristics were obtained. The value of the different parameters in the different genotypes is presented in Table 6. This Table shows that the subjects carrying Arg64 in the homozygous state had a greater BMI, waist and hip circumference, W/H ratio, cholesterol, triglyceride, LDL-C, and plasma leptin, insulin, and glucose compared with those with the Trp64Trp and Trp64Arg genotypes, and the difference was significant. However, the decrease in HDL level was not statistically significant (*p*-value = 0.335) in individuals with different ADRB3 genotypes (Table 6).

**Table 5:** The genotypes and allele frequencies of ADRB3 Trp64Arg polymorphism in control, overweight and obese subjects

*Genotype*	*Control* *T = 115*	*Overweight* *T = 68*	*Obese* *T = 146*	*χ* ^*2*^ *Odds ratio (95% CI)*	*P- value*
Genotype Frequency					
Trp\Trp	112 (97.4)	61 (89.7)	116 (79.5)	***χ*** ^*2*^ *OW = 4.9* *Ob = 12.4* *Odds ratio (95% CI) Control* vs *OW* *4.3 (1–17)* *Odds ratio (95% CI) Control* vs *Obese* *7 (2–24)*	*P value for Odds ratio in normal* vs *overweight* *0.02* *and normal* vs *obese:* *0.0004*
Trp\Arg	3 (2.6)	7 (10.3)	23 (15.8)		
Arg\Arg	0	0	7 (4.8)		
Allele Frequency					
Trp64	98.7%	94.9%	87.3%	*Odds ratio (95% CI) Control* vs *OW* *4.11 (1.04–16.1)* *Odds ratio (95% CI) Control* vs *Obese 10.98 (3–36)*	*P* = 0.02
Arg64	1.3%	5.1%	12.7%		*P* = 0.00001

OW = Overweight; Ob = Obese; CI: Confidence interval; ***χ***^***2***^ = Chi square

**Table 6:** Phenotypic Characteristics of subjects grouped according to ADRB3 polymorphism at Codon 64

Phenotypic variables	Trp64Trp64 (*n* = 289) *mean ± SE*	Trp64Arg64 (*n* = 33) *mean ± SE*	Arg64Arg64 (*n* = 7) *mean ± SE*	P-value
BMI (kg/m^2^)	27.640± 0.402	32.48± 6.36	45.4± 1.8	0.0001
Waist(cm)	86.51± 1.06	98.24± 2.95	119.14 ± 4.83	0.0001
Hip(cm)	106.82± 0.88	112.18± 2.55	129.57± 3.72	0.0001
W/H ratio	0.81 ± 0.01	0.87 ± 0.02	0.92 ± 0.04	0.0001
Cholesterol (mmol/L)	3.94± 0.05	4.36± 0.165	5.63± 0.32	0.0001
Triglyceride (mmol/L)	1.02± 0.03	1.31± 0.09	1.643± 0.181	0.0001
HDL (mmol/L)	1.193 ± 0.02	1.12± 0.05	1.08± 0.120	0.335
LDL (mmol/L)	2.051± 0.045	2.319± 0.147	3.286± 0.244	0.0001
Leptin (ng/ml)	21.95± 1.063	24.38± 2.52	57.14± 10.74	0.0001
Fasting Insulin (pmol/L)	79.018± 2.24	101.6± 6.88	111.70± 17.37	0.001
Fasting Glucose(mmol/L)	4.903± 0.030	5.234± 0.12	4.89± 0.06	0.003

Values are mean ± SEM. *Abbreviations*: *BMI*, body mass index; *W/H*, waist hip ratio

## Discussion

This is the first study to report the relationship between *ADRB1* and *ADRB3* gene polymorphism and obesity-related phenotypes in the Saudi population. Since in our previous report, we had observed an association between Gln27Glu polymorphism of *ADRB2* gene, obesity, and other related disorders in Saudi population [9] our interest was to explore if any association existed between *ADRB1* (rs1801253**)** and *ADRB3* (rs4994) and these abnormalities. Hence, during this study, a large cohort of 329 subjects was genotyped for Gly389Arg in *ADRB1* (rs1801253**)** and Trp64Arg in *ADRB3* (rs4994) genes and the association of the variant allele with metabolic parameters were analyzed. The results of our investigation have clearly highlighted the strong association with *ADRB3* polymorphism, where the mutant Arg allele in *ADRB3*, significantly associates with a greater body weight and higher BMI, elevated leptin and dyslipidaemias both in heterozygous and homozygous. It also results in elevated blood glucose level and hyperinsulinaemia. However, the *ADRB1* polymorphism Arg389Gly, shows no association either with the BMI or related disorders. The *ADRB1* is a candidate gene for obesity due to its role in catecholamine mediated energy homeostasis. In 2008, Ohshiro and coworkers had shown a strong association between mutations in the *ADRB1* and massive obesity in Japanese [56]. In obese individuals, the degree of weight loss during a very low calorie diet has been shown to correlate with changes in ADRB1 protein concentration in adipose tissue [57, 58]. An investigation involving a population cohort of 761 women indicated that women carrying the Gly49 genotype had greater elevation in BMI over 15 years compared to those with the Ser49 genotype [59]. Dionne et al., [7] reported that Gly389Arg exhibited a strong relationship with obesity in Caucasian women. In contrast, some studies have reported the Arg389Gly polymorphism had no significant relationship to obesity in Danish [21], Swedish [60] and Japanese [56] subjects, suggesting that it has no role in human obesity. Masuo and Lambert [10] have reviewed various studies about the relationship of polymorphism in either Gly49-Gly389 or Ser49Gly- Arg389Gly in *ADRB1* with obesity or obesity related disorders and have reported contradicting findings among different populations which means that the relationship of *ADRB1* polymorphism to obesity could vary between different populations. In fact, our findings for Arg389 Gly polymorphism in *ADRB1* amongst Saudi population are in line with earlier published reports [7, 21, 61, 62] and suggest that *ADRB1* Arg389Gly polymorphisms do not contribute to obesity and related disorders in the Saudi population.

The *ADRB3* gene is expressed in adipose tissues and stimulates the mobilization of lipids from the WAT and increases thermo-genesis in BAT [10]. Mutation of *ADRB3* in WAT could slow lipolysis and thereby cause the retention of lipids in adipocytes and may contribute to visceral obesity in humans. Interestingly, extensive studies have investigated the role of Trp64Arg (rs4994) in the development of obesity, T2DM, hypertension (HT), cardiovascular disease CAD, dyslipidaemias. Table 7 summarizes some of the studies reported from different populations and shows that there are extensive contradictions between populations, between genders and within different female age groups. Some studies suggest a strong relationship between obesity and Trp64Arg polymorphism in some populations [20, 26–28, 31–33, 35, 37–40, 45, 48, 51, 61–65]. Whereas, other studies on same or different populations have failed to show any association [21, 36, 41, 46, 49, 50, 52]. Even within the same population there are contradictory reports (Table 7). This makes the study of Trp64Arg polymorphism necessary in every population. The present study has found a strong relationship between obesity and Trp64Arg polymorphism in the Saudi population. This is a similar association that we have shown earlier to exist between Gln27Glu polymorphism in *ADRB2* gene and obesity related disorders such as hypertriglyceridemia, hyperinsulinemia, and hyperleptinemia in Saudis [9].

**Table 7:** Studies reporting the association of Trp64Arg (rs4994) with obesity and obesity-related disorders in different populations

Population	Study showed rs4994:	References
Asians	Association with T2DM (meta-analysis)	[26]
Non-Asians	No association	
Asians	Association with BMI (meta-analysis)	40
African American	No association with BMI	52
S. African women	No association with MS	41
Brazilians/Caucasians	Combined effect in the modulation of OW/obesity and HDL-C in T2DM	31
Balinese	Association with obesity in rural females only	38
Caucasians	Association with elevated BMI and BMD	48
Chile (Ayara natives)	No association with BMI	50
Chinese	Association with HT and dyslipidaemias (metaanalysis)	27
Hungarian children	Association with increase body weight and BMI	28
S. Italians	Insulin resistance in males only	46
Japanese	Association with increase BMI	51
	No association with BMI	49
	No relation with MS	45
Japanese children	Association with increases visceral fat, blood pressure, triglycerides and reduced TAG	32
Japanese men	Association with annual weight gain	35
Kashmiris	Association with increase BMI, W/H ratio, dyslipidaemias, uncontrolled T2DM	53
Kyrgy	Association with increase obesity, abdominal obesity, decreased HDL	37
Mexican	Association with increase T2DM and MS	39, 29
Russians	Association with increase fat, glucose level, and uric acid	33
S. Spain	Association with T2DM	44
Taiwanese	No association with obesity	36
Saudis	Obesity, hyperlipidaemias, hyperinsulinaemia, hyperleptinaemia.	This study

The *ADRB2* and *ARDB3* gene polymorphisms may act as predictive markers for obesity and obesity related disorders in Saudis.

## Conclusion

Our present results shows for the first time that obesity and related disorders in Saudis are linked to polymorphism of Trp64Arg (rs4994) in *ADRB3* gene but not to Arg389Gly (rs1801253) in *ARDB1* gene.

## Abbreviations

ADRB1: β1-adrenoceptor, β3
ADRB2: β2 beta-adrenoceptor
ADRB3: β3 beta-adrenoceptor
Arg: Arginine
BAT: Brown adipose tissue
BMI: Body mass index
CAD: Cardiovascular disease
CI: 95% confidence interval
EDTA: Ethylenediaminetetraacetic acid
Gly: Glycine
HDL: High-density lipoprotein
HT: Hypertension
kg/m^2^: Kilogram/m square
LDL: Low-density lipoprotein
mmol/L: `Millimol/l
ng/ml: Nanogram/ml
Ob: Obese
OR: Odds ratio
OW: Overweight
PCR: Polymerase chain reaction
pmol/L: Picomol/l
r: Pearson’s correlation coefficient
RIA: Radioimmunoassay
SEM: Standard Error of the Mean
SNP: Single nucleotide polymorphism
T2DM: Diabetes mellitus Type 2
Trp: Tryptophan
UCP1: Uncoupling protein-1
WAT: White adipose tissue
WHR: Waist-to-hip ratio
